# Identifying subtypes of longitudinal motor symptom severity trajectories in early Parkinson’s disease patients

**DOI:** 10.3389/fneur.2025.1597132

**Published:** 2025-08-20

**Authors:** Xiaozhou Xu, Shushan Zhang, Chuanying Xu, Wei Zhang, Hui Zhao, Yumeng Liu, Shilei Zhai, Jie Zu, Zhining Li, Lishun Xiao

**Affiliations:** ^1^Department of Biostatistics, School of Public Health, Xuzhou Medical University, Xuzhou, China; ^2^Office of Hospital Quality and Safety Management, The First People’s Hospital of Lianyungang, Lianyungang, China; ^3^Department of Ultrasound, The Fifth Affiliated Hospital of Sun Yat-sen University, Zhuhai, China; ^4^Department of Neurology, The Affiliated Hospital of Xuzhou Medical University, Xuzhou, China; ^5^Department of Neurology, The First Clinical College, Xuzhou Medical University, Xuzhou, China; ^6^Department of Neurology, The Second Affiliated Hospital of Xuzhou Medical University, Xuzhou, China; ^7^Center for Medical Statistics and Data Analysis, Xuzhou Medical University, Xuzhou, China; ^8^Key Laboratory of Human Genetics and Environmental Medicine, Xuzhou Medical University, Xuzhou, China

**Keywords:** Parkinson’s disease, motor symptoms, longitudinal trajectory, unsupervised clustering, machine learning

## Abstract

**Background:**

Motor symptoms of Parkinson’s disease (PD) patients affect their ability of daily activities. Identifying distinct trajectories of motor symptom progression in PD patients can facilitate long-term management.

**Methods:**

A total of 155 PD patients were acquired from the Parkinson’s Disease Progression Marker Initiative (PPMI). Distinct longitudinal trajectory clusters of motor symptom progression in PD patients were identified by unsupervised self-organizing maps (SOMs), and baseline characteristics were compared among different clusters. Linear mixed-effect analysis was utilized to estimate the longitudinal courses of some cardinal motor symptoms among clusters, while survival analysis was used to compare time-to-clinical milestones within 5 years. The support vector machine (SVM) was built to predict patients’ trajectory clusters, and its performance was evaluated through the mean area under the receiver-operating characteristic curve (mAUC), accuracy and macro *F*_1_-score. Shapley values were calculated to interpret individual variability.

**Results:**

The optimal clusters by SOMs are 3. Cardinal motor symptoms of the progressive cluster worsened more rapidly, and this cluster is more likely to have impaired balance, loss of independence, sleep disturbance, and cognitive impairment within 5 years. The mAUC, accuracy, and macro *F*_1_-score of multi-class SVM model were 0.8846, 0.7692, and 0.7778, respectively. An interactive web application was developed to predict the individual’s trajectory cluster.

**Conclusion:**

Subtyping motor symptom progression into different trajectories can improve patients’ management. Using baseline data to predict which trajectory cluster a patient belongs to may help develop interventions.

## 1 Introduction

Parkinson’s disease (PD) is a progressive neurodegenerative disorder, clinically manifested by typical motor symptoms and a variety of nonmotor symptoms (1). As a chronic disease that requires long-term management, motor symptoms not only negatively impact PD patients’ daily activities and quality of life (2), but also serve as prognostic factors for rapid decline in PD-related disability (3). Despite its ubiquity, the heterogeneity of motor symptom progression trajectories in early PD patients remains yet to be explored (4). Rational subtyping early PD patients offers practical benefits for clinicians, patients, and caregivers. Understanding the progression trajectory of motor symptoms in PD patients allows for prognosis estimation, which aids clinicians in devising personalized treatment plans and follow-up schedules. It can also provide supportive counseling to patients and their families, helping them maintain a positive outlook on life. Additionally, including PD patients with more rapid progression of motor symptoms as an enrichment factor in clinical trials can potentially reduce the required sample size, inform the design, lower costs, and enhance the clinical trials’ sensitivity to detect treatment effects.

The conventional method for classifying motor subtypes of PD is to divide patients into tremor-dominant and non-tremor-dominant subtypes based on the most prominent clinical symptoms (1, 5–7), which is a hypothesis-driven approach. However, these subtype classification methods only explain the current heterogeneity of motor symptoms of PD patients, and the reliability of the subtype is questioned (8). Since subtypes may shift as the disease progresses (9), motor subtypes tend to be more heterogeneous early in the disease process, converging towards a common subtype as the disease progresses (10). Theoretically, the progression of such motor subtypes is orthogonal to the progression of the PD itself (11, 12). Therefore, longitudinal data should serve as the basis for identifying motor subtypes, not merely for evaluating the trends of progression between different subtypes.

A more realistic representation of the disease course requires a combination of a data-driven schema. Data-driven cluster analysis has the potential advantage of requiring no prior assumptions (13), and can provide more information for the understanding of complex mechanisms. Previous studies used cluster analysis to define clinical PD subtypes (14–17), but most had significant methodological disadvantages (18). Because data-driven methods are highly sensitive to the variables chosen for clustering, the results with different variables can be quite heterogeneous and controversial (19). Therefore, the selection of variables should be determined based on specific research purposes and assess the quality of clusters for clinical relevance. Additionally, several studies are limited by approaches that only provide descriptions at the group level and are unable to assign new individual patients to subtypes, which makes it difficult to apply the findings to individual patients. Given that the symptoms, treatments, and treatment responses in PD patients change over time, relying solely on hypothesis-driven methods or data-driven methods may not be sufficient. To meet the needs of patients, caregivers, clinicians, and researchers, new approaches are needed to describe the heterogeneity of PD patients from the perspective of disease progression. Based on the purpose-driven framework recently proposed by the Movement Disorder Society (MDS) (18), which emphasizes considering the purpose of use when developing and applying PD subtypes, we distinguished PD patients with different motor symptom progression trajectories, and defined subtypes through this heterogeneity in progression trajectories. We believe that this approach of using progression trajectories to define subtypes reflects disease progression and may be more appropriate for progressive disease.

Movement Disorders Society Sponsored revision of Unified Parkinson’s Disease Rating Scale (MDS-UPDRS) is a single tool for assessing specific aspects of PD globally, Part III of the motor examination can reliably assess the severity of objective motor symptoms (20, 21). To define trajectory clusters based on the evolution of motor symptoms over time, we used the MDS-UPDRS-Part III score to represent motor symptom severity at different follow-up visits for PD patients and applied unsupervised self-organizing maps (SOMs) to identify distinct motor trajectory clusters in early PD patients. Considering the clinical applicability of the trajectory clusters, we then constructed a machine learning (ML) model using baseline clinical data to assign new individual patients to their respective clusters.

The aims of this study were to: (1) identify distinct progression trajectories of motor symptoms in early PD patients; (2) explore differences in baseline clinical biomarkers among different trajectory clusters; (3) compare the progression rates of cardinal motor symptoms and the proportions of patients reaching key clinical milestones among different trajectory clusters during a five-year follow-up; (4) develop an interactive application based on an ML model to assign new individual patients to their respective clusters.

## 2 Methods

### 2.1 Participants

We enrolled 155 *de novo* drug-naïve PD patients from the Parkinson Progression Markers Initiative (PPMI), an international, multicenter, prospective, observational study (18). Participants in the PPMI cohort were followed longitudinally for clinical, imaging, and biospecimen biomarker assessment using standardized data acquisition protocols at 21 clinical sites. The study was approved by the Institutional Review Board of all participating sites, and written informed consent was obtained from all participants before inclusion in the study. The inclusion criteria for this study were as follows: (1) baseline clinical data available, (2) a drug-naïve PD diagnosis of Hoehn and Yahr (H&Y) stages I–II, and (3) complete MDS-UPDRS-Part III data from baseline to 5 years of follow-up. The detailed flowchart is shown in Supplementary Figure 1.

Accelerating Medicines Partnership Parkinson’s Disease (AMP PD) aims to identify and validate biomarkers related to the diagnosis, prognosis and progression of PD, and to develop new approaches for improving clinical trial design and treatment. Given that the AMP PD dataset incorporates PPMI cohort data and only partial follow-up information for us is accessible through this composite resource, distinguishing PPMI patients form AMP PD becomes challenging. Consequently, AMP PD data is only used to verify trajectory clustering. More information can be found at: http://ppmi-info.org/ and https://amp-pd.org/.

### 2.2 Assessment of clinical information

The motor and non-motor assessments were completed by all participants at the baseline visit. Included participants underwent common PD tests such as the H&Y stage and MDS-UPDRS. The MDS-UPDRS total score is the sum of parts I to III of MDS-UPDRS, which include non-motor experiences in daily life (Part I); motor experiences in daily life (Part II) and motor examination (Part III) (22). Daily living ability was assessed using the modified Schwab & England activities of daily living (ADL) score.

Assessment of non-motor symptoms includes autonomic tests, neurobehavioral tests, neuropsychological tests, olfactory tests, and sleep disorder tests. Autonomic tests include Scales-for-outcomes-in-Parkinson’s disease-autonomic (SCOPA-AUT) score. Neurobehavioral tests include Geriatric Depression Scale (GDS) score, Questionnaire for Impulsive-Compulsive Disorders in Parkinson’s Disease (QUIP) and the State-Anxiety Index (STAI) score. Neuropsychological tests include Benton Judgment of Line Orientation (JoLO) Test, Hopkins Verbal Learning Test (HVLT), Letter Number Sequencing (LNS) score, Montreal Cognitive Assessments (MoCA) score, Symbol Digit Modalities Test (SDMT), Semantic Fluency Total (SFT) Score. Olfactory tests include University of Pennsylvania Smell Identification Test (UPSIT). Sleep disorder tests include Epworth Sleepiness Scale (ESS) score, and rapid eye movement sleep behavior disorder (RBD) is evaluated using the REM Sleep Behavior Disorder Screening Questionnaire (RBDSQ) score.

The dopamine transporter (DAT) binding rate (caudate and putamen) and cerebrospinal fluid (CSF) proteins (α-synuclein, Aβ_1–42_, t-Tau, p-Tau_181_) were collected. The details of the DAT processing and cerebrospinal fluid biomarker measurements could be found in Supplementary material.

### 2.3 Statistical analysis

All analyses were performed using R version 4.2.3 statistical software, with missing values of the independent variables imputed by the “DMwR2” package. The independent variables with missing values in the baseline data were DaTScan mean caudate SBR, DaTScan mean putamen SBR, CSF Aβ_1–42_, CSF α-synuclein, CSF T-Tau, CSF P-Tau_181_, with missing rates of 1.29, 1.29, 3.23, 1.94, 3.87, and 7.74%, respectively. These rates met the criteria for imputation, and missing values were imputed using the k-nearest neighbors method in the “DMwR2” package, with the parameter (*n*) set to 20, and impute before data normalization.

Each patient’s baseline and five-year follow-up MDS-UPDRS-part III score represent the longitudinal trajectory of the patient’s motor symptom severity. We conducted the unsupervised SOM to identify distinct clusters of individual trajectories using the “som” package. As a preprocessing step, the data was normalized to scale-free values. Hyperparameter optimization was then performed before clustering, the learning rates started from 1.00 and was set to 0.90 for ordering and to 0.02 for tuning, and a neighborhood distance was set at 1.00 with hexagonal topology (23). Given the topological preservation properties of SOMs and their conceptual alignment with k-means clustering method (23), the best number of clusters was determined using the “NbClust” package, which provides 26 fit indices. The best fit was selected based on a plurality of these indices (24). The same preprocessing steps and clustering methods were applied to AMP PD.

Baseline demographic characteristics, clinical assessments, cerebrospinal fluid biomarkers, and neuroimaging results were compared between trajectory clusters. For continuous variables, normality distribution was measured using Shapiro–Wilk’s test. Mean and standard deviation (SD) were used to describe central tendency and dispersion if the continuous variable was normally distributed, otherwise median and interquartile range (IQR) were used. Normal distributed continuous variables with homoskedasticity which was measured by Levene’s test, the means among three groups were compared by analysis of variance (ANOVA), and a post-hoc test was performed using Tukey’s method. For non-normally distributed continuous variables, Kruskal–Wallis’s test was used to compare the medians among groups, and a post-hoc test was performed using Dunn’s method. The categorical variables were described by frequency and constituent ratio, and the differences among groups were compared by chi-square test or Fisher’s test. The level of statistical significance was predefined as 0.05 (two-sided).

Linear mixed-effect model was utilized to estimate the longitudinal courses of some cardinal motor symptoms between clusters via the “lme4” package. The core motor symptoms are assessed based on the sum of the corresponding MDS-UPDRS subitems (25), namely: tremor, sum of MDS-UPDS subitems 2.10, 3.15–3.18; postural instability with gait disorder (PIGD), sum of MDS-UPDRS subitems 2.12–2.13, 3.10–3.12; bradykinesia, sum of MDS-UPDRS subitems 3.5–3.8, 3.14; rigidity, MDS-UPDRS subitem 3.3.

Survival analyses were performed to compare clinical milestones among different trajectory clusters via the “survival” package. The following time to clinical milestones times were assessed up to the follow-up year 5: (1) H&Y score ≥3, indicating at least the presence of balance impairment with mild to moderate disease severity (loss of recovery from a retropulsive stress); (2) Modified Schwab & England ADL score <80%, corresponding to a threshold of not being completely independent in performing daily activities; (3) RBDSQ score ≥3, corresponding to a cutoff for diagnosis of RBD Positive; (4) MoCA score ≤23, corresponding to a cutoff for diagnosis of cognitive impairment.

### 2.4 Construction and explanation of the ML model

The baseline data was randomly divided into a training set (75%) and a test set (25%). The training set was utilized for both parameter tuning and final model development. Through grid search within a predefined hyperparameter space, the optimal hyperparameter combination was identified. During this process, five-fold cross-validation was implemented to mitigate random sampling bias. This approach minimizes the mean prediction error while ensuring high classification accuracy and effectively avoiding overfitting. The independent test set was subsequently employed to evaluate the model’s predictive performance.

We utilized a classification support vector machine (SVM) model using baseline clinical data of PD patients. The evaluation indicators of the model performance included the mean area under the receiver-operating characteristic curve (mAUC), accuracy, macro-average sensitivity, macro-average specificity and macro *F*_1_-score.

In addition, considering the heterogeneity of PD patients, we calculated Shapley values for each patient’s characteristics with the “iml” package. Shapley value computes feature contributions for individual prediction, which fairly distributes the difference of the instance’s prediction and the datasets average prediction among the features. The SVM model was finally adopted to develop an interactive web application with the “shiny” package. After inputting the patient’s characteristics, the tool outputs the predicated trajectory clusters and the Shapley value plot.

## 3 Results

### 3.1 Baseline characteristics

A total of 155 drug-naïve patients with PD were included. The median (IQR) of the age was 61.51 (12.11) years, 106 (68.39%) were male patients, and the median (IQR) of disease duration was 3.87 (4.60) months. There were 92 (59.35%) patients with H&Y stage 1 and 63 (40.65%) patients with H&Y stage 2 at baseline (see Supplementary Table 1).

### 3.2 Trajectory clusters description

**Figure 1:**
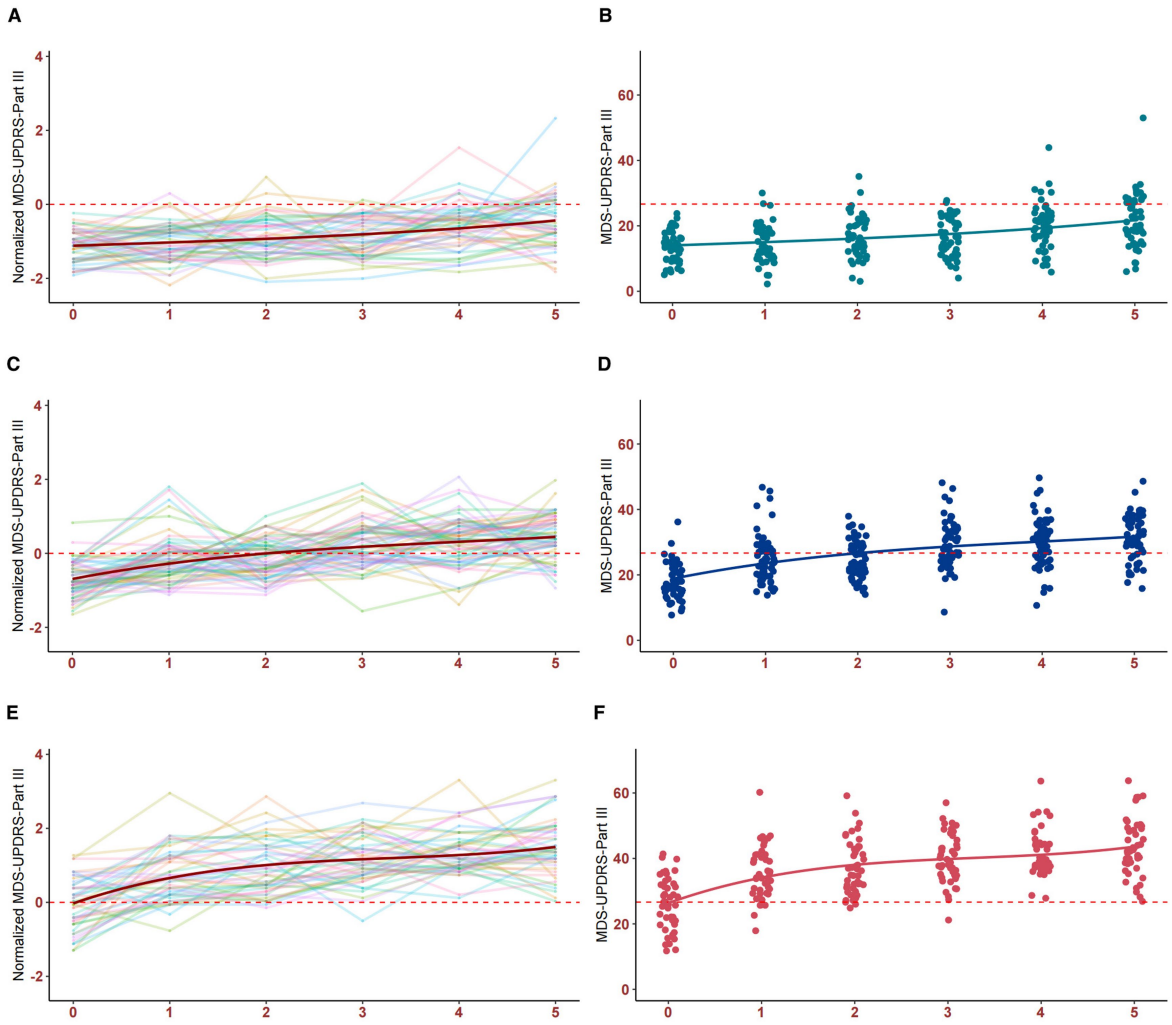
SOMs of MDS-UPDRS-Part III score trajectory per cluster among patients. Individual data points indicate the MDS-UPDRS-Part III score for each patient. The trendline shows the mean MDS-UPDRS-Part III score at each follow-up year. Individual motor symptom severity traces clustered by SOMs from each patient represented as scale-free normalized values for stable cluster **(A)**, intermediate cluster **(C)**, progressive cluster **(E)**, and as non-normalized values for stable cluster **(B)**, intermediate cluster **(D)**, progressive cluster **(F)**.

Out of 26 fit indices, 13 indices suggested three trajectory clusters was the optimal solution. The baseline MDS-UPDRS-Part III score of the cluster 3 (26 points) was significantly higher than those of cluster 1 (14 points) and cluster 2 (18 points) (see Table 1). Considering that the total participants showed an annual increase of approximately 2.34 points in MDS-UPDRS-Part III score, thus three trajectory clusters that can be interpreted as the stable cluster [Cluster 1, *N* = 50 (32.26%), annual increase of 1.51 points], the intermediate cluster [Cluster 2, *N* = 60 (38.71%), annual increase of 2.46 points], and the progressive cluster [Cluster 3, *N* = 45 (29.03%), annual increase of 3.11 points], as shown in Figure 1.

**Table 1:** Baseline demographic and clinical variables among three trajectories.

Characteristic	Stable cluster (*N* = 50)	Intermediate cluster (*N* = 60)	Progressive cluster (*N* = 45)	*p*-value
Age, years	58.7 [12.29]	60.93 [11.23]	63.95 [10.13]	**0.030**^b^
Gender, *n* (%)				
Male	32 (64.00)	36 (60.00)	38 (84.44)	**0.021**
Female	18 (36.00)	24 (40.00)	7 (15.56)	
Duration, months	3.55 [3.89]	4.13 [4.48]	3.70 [5.03]	0.982
Education, years	15 [3]	16 [4]	16 [4]	0.066
MDS-UPDRS total score	21 [8.75]	28 [15.25]	38 [11]	**<0.001**^a^^,^^b^^,^^c^
MDS-UPDRS-Part I score	3 [4]	5 [6]	6 [4]	**<0.001**^a^^,^^b^^,^^c^
MDS-UPDRS-Part II score	3 [3.75]	4 [6]	7 [4]	**<0.001**^a^^,^^b^^,^^c^
MDS-UPDRS-Part III score	14 [8]	18 [7.25]	26 [12]	**<0.001**^a^^,^^b^^,^^c^
Rigidity	2 [2]	3 [3]	6 [3]	**<0.001**^a^^,^^b^^,^^c^
Tremor	4 [4]	5 [4]	5 [5]	0.823
Bradykinesia	4 [4]	5.5 [4]	9 [7]	**<0.001**^a^^,^^b^^,^^c^
PIGD	0 [1]	1 [0.5]	1 [1]	**<0.001**^a^^,^^b^^,^^c^
H&Y stage, *n* (%)				**<0.001**
Stage I	40 (80.00)	37 (61.67)	15 (33.33)	
Stage II	10 (20.00)	23 (38.33)	30 (66.67)	
Schwab-England ADL score	95 [10]	90 [10]	90 [0]	**0.001**^b^^,^^c^
SCOPA-AUT score	6 [6.75]	8 [6.25]	10 [5]	**0.009**^b^
GDS score	1 [3]	2 [2.25]	2 [2]	0.135
QUIP score	0 [0]	0 [1]	0 [0]	0.214
STAI total score	61 [26.25]	62 [22.25]	60 [30]	0.781
STAI state subscore	30 [14]	30 [11.25]	32 [18]	0.550
STAI trait subscore	29.5 [10.75]	31.5 [11]	32 [13]	0.496
JoLO score	14 [3]	13 [3]	14 [2]	0.515
HVLT Discrimination Recognition	10 [2]	10 [2]	10 [1]	**0.036**^b^^,^^c^
HVLT Immediate/Total Recall	25.24 ± 4.42	25.00 ± 4.95	24.24 ± 4.66	0.563
HVLT Retention	0.90 [0.20]	0.89 [0.26]	0.88 [0.27]	0.473
HVLT False Alarms	1 [1]	1 [2]	1 [1]	0.172
HVLT Delayed Recall	9 [3]	9 [3.25]	8 [3]	0.641
HVLT Delayed Recognition	12 [1]	12 [1]	11 [2]	0.233
LNS score	11.5 [3.75]	11 [3.25]	10 [3]	0.054
MOCA score	28 [2]	27 [2.25]	27 [3]	0.065
ESS score	4 [3.75]	5 [4]	4 [4]	0.392
RBDSQ score	4 [3]	3 [3]	4 [5]	0.167
SDMT score	44 [11.75]	46 [12.25]	39 [14]	**0.006**^b^^,^^c^
SFT score	51 [18.5]	48.5 [16.75]	44 [13]	**0.040**^b^^,^^c^
SFT animal subscore	22 [6.75]	21 [8.25]	19 [7]	0.110
SFT fruit subscore	13 [4.75]	14 [6]	13 [6]	0.711
SFT vegetable subscore	15.26 ± 4.80	15.23 ± 4.36	12.87 ± 3.69	**0.009**^d^^,^^e^
UPSIT score	25 [11.75]	23.5 [16]	19 [13]	**0.032**^b^
DaTScan mean caudate SBR	2.03 ± 0.51	2.02 ± 0.49	1.78 ± 0.45	**0.021**^d^^,^^e^
DaTScan mean putamen SBR	0.83 [0.35]	0.80 [0.29]	0.64 [0.31]	**0.002**^b^^,^^c^
CSF Aβ_1–42_ (pg/mL)	927 [612.58]	896.5 [480.48)	876.7 [369.5]	0.682
CSF α-synuclein (pg/mL)	1425.37 [677.50]	1436.95 [841.60]	1373.70 [562.55]	0.625
CSF T-Tau (pg/mL)	163.25 [72.67]	153.45 [71.87]	150.52 [36.90]	0.851
CSF P-Tau_181_ (pg/mL)	13.27 [5.85]	13.30 [4.99]	12.66 [3.81]	0.693

Descriptive statistics for continuous variables are presented as median [IQR] or mean ± SD.

a Stable cluster is significantly different from intermediate cluster (Dunn’s method with *p* < 0.05).

b Stable cluster is significantly different from progressive cluster (Dunn’s method with *p* < 0.05).

c Intermediate cluster is significantly different from progressive cluster (Dunn’s method with *p* < 0.05).

d Stable cluster is significantly different from progressive cluster (Tukey’s method with *p* < 0.05).

e Intermediate cluster is significantly different from progressive cluster (Tukey’s method with *p* < 0.05).

*p*-values less than 0.05 are given in bold.

To ascertain the stability of the trajectory clustering, we utilized data from the AMP PD as a validation set, and similarly observed the existence of three distinct progressive trajectories in the motor symptoms of PD patients (see Supplementary Figure 2).

### 3.3 Comparison of baseline demographics and clinical variables among three trajectory clusters

As illustrated in Table 1, there were no significant differences among three clusters in terms of disease duration and years of education. Compared with the stable cluster, patients in the progressive cluster were older (*p* = 0.030), and there were differences in gender (*p* = 0.021) and H&Y stage (*p* < 0.001) among the three clusters (see Table 2).

**Table 2:** Annual change of motor function scores in trajectory clusters.

Annual change	Total	Stable cluster (*n* = 50)	Intermediate cluster (*n* = 60)	Progressive cluster (*n* = 45)
MDS-UPDRS-Part II, score	0.759	0.606	0.734	0.962
MDS-UPDRS-Part III, score	2.344	1.508	2.463^*^	3.113^***^
Rigidity, score	0.549	0.437	0.500	0.737^*^
Tremor, score	0.358	0.185	0.325	0.595^*^
Bradykinesia, score	0.838	0.537	0.997^**^	0.958^*^
PIGD, score	0.241	0.149	0.214	0.370^**^
Modified Schwab & England ADL, score	−1.569	−1.106	−1.643	−1.984^*^

The symbol * indicates a statistically significant difference compared to the stable cluster, and * represents *p*-value < 0.05, ** represents *p*-value < 0.01, and *** represents *p*-value < 0.001.

The progressive cluster had the highest MDS-UPDRS total score, the highest MDS-UPDRS-Part I, Part II, Part III, rigidity, bradykinesia, PIGD and SCOPA-AUT scores, and the worst modified Schwab & England ADL, HVLT Discrimination Recognition, SDMT, SFT and UPSIT scores at baseline. Conversely, the stable cluster exhibited the lowest severity of motor and non-motor manifestations with the least impaired core motor symptoms, neuropsychological features, autonomic and olfactory dysfunctions at baseline. For almost all manifestations, PD patients of the intermediate clusters had values intermediate between the stable cluster and the progressive cluster.

In addition, the DaTScan mean caudate SBR and the DaTScan mean putamen SBR in the stable cluster and the intermediate cluster were significantly higher than those in the progressive cluster at baseline. No significant difference was observed among the three clusters for CSF biomarkers at baseline.

### 3.4 Annual change of motor function scores in trajectory clusters

In terms of some cardinal motor symptoms progression, the annual decline of MDS-UPDRS-Part III, rigidity, tremor, bradykinesia, PIGD, and modified Schwab & England ADL scores of the progressive cluster were significantly faster than those of the stable cluster. The annual decline in scores of MDS-UPDRS-Part III and bradykinesia of the intermediate cluster were also significantly faster than the stable cluster.

### 3.5 Survival analysis of reaching key clinical milestones

**Figure 2:**
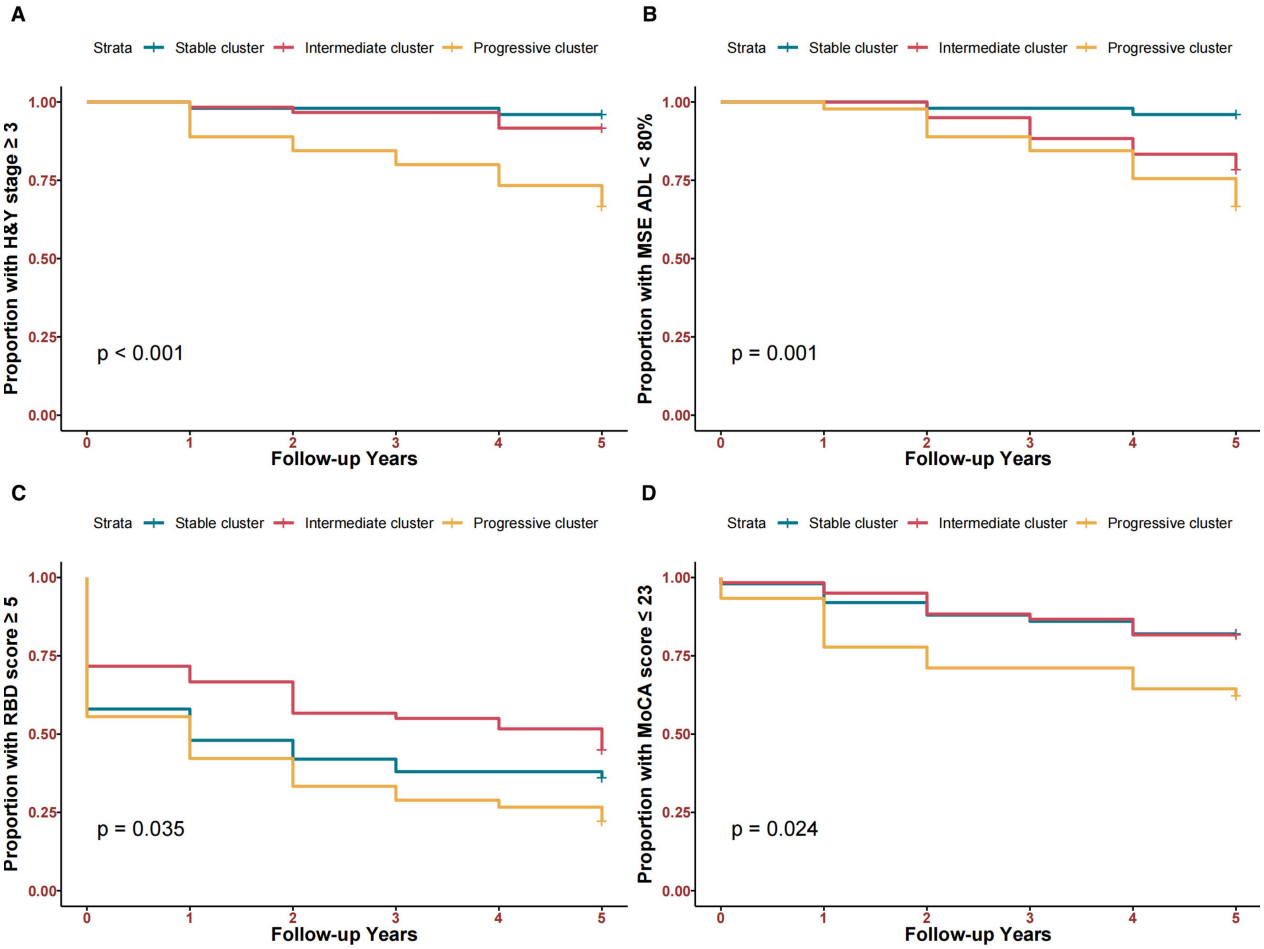
Kaplan–Meier curves comparing time-to-clinical milestones among three clusters within 5 years. **(A)** Time to H&Y stage ≥3. **(B)** Time to Modified Schwab & England ADL <80%. **(C)** Time to RBD score ≥5. **(D)** Time to MoCA score ≤23.

The progressive cluster had a higher chance of reaching key clinical milestones within 5 years follow-up. 14.2% of participants reached H&Y ≥3 (4.0% for the stable cluster, 8.3% for the intermediate cluster, and 33.3% for the progressive cluster) (Figure 2A). 19.4% of participants reached modified Schwab & England ADL <80% (4.0% in the stable cluster, 21.7% in the intermediate cluster, and 33.3% in the progressive cluster) (Figure 2B). 64.5% of participants reached RBDSQ ≥5 (64.0% in the stable cluster, 55.0% for the intermediate cluster, and 77.8% for the progressive cluster) (Figure 2C). 23.9% of participants reached MoCA ≤23 (18.0% in the stable cluster, 18.3% in the intermediate cluster, and 37.7% in the progressive cluster) (Figure 2D).

### 3.6 Treatment response

In the early stages of PD, the progressive cluster is more likely to show symptom improvement after dopaminergic drug treatment. Over 75% of patients demonstrated improvement in MDS-UPDRS-Part III scores after dopaminergic drug intake compared to the off state during the five-year follow-up period (Supplementary Figure 3).

### 3.7 Machine learning model and interactive web application

Before establishing the machine learning model, features with variance close to zero such as race were excluded. Subsequently, features with correlations higher than 0.8, such as diagnostic age and DaTScan mean putamen SBR, were excluded. Finally, the recursive feature elimination method was adopted to further optimize the feature set.

The following prediction features are finally selected: age, gender, years of education, family history, disease duration, most affected side, H&Y stage, motor subtype, tremor, rigidity, bradykinesia, postural instability with motor disorder, activities of daily living, MDS-UPDRS-Part I score, MDS-UPDRS-Part II score, MDS-UPDRS-Part III score, MDS-UPDRS total score, UPSIT score, JoLO score, ESS score, GDS score, HVLT Discrimination Recognition, HVLT Immediate/Total Recall, HVLT Retention, HVLT False Alarms, HVLT Delayed Recall, HVLT Delayed Recognition, LNS score, QUIP Score, RBDSQ score, SCOPA-AUT score, SFT animal subscore, SFT fruit subscore, SFT vegetable subscore, SFT score, STAI Total score, STAI state subscore, STAI trait subscore.

We developed a multi-class SVM model to identify patient’s motor trajectory cluster using baseline demographic and clinical variables (mAUC = 0.8846, accuracy = 0.7692, macro-average sensitivity = 0.7866, macro-average specificity = 0.8842, macro *F*_1_-score = 0.7732).

Given the individual differences in PD patients, we calculated feature contributions using the Shapley value. Shapley value distributes the difference between the individual prediction and the average prediction to each feature. As shown in Supplementary Figure 4, we interpret the instance using Shapley values with the progressive cluster as the target. The actual prediction denotes the predicted ending, 1 for the progressive cluster and 0 for not the progressive cluster. Vertical coordinates denote the features of the instance. The horizontal coordinate denotes the phi value of the corresponding feature, where a larger phi value indicates a larger contribution for this instance compared to the average prediction of the dataset. For this instance, the high predictive value of the patients mainly stems from poorer MDS-UPDRS scores, being female, better Semantic fluency, etc., while features such as years of education and no family history of PD partially offset this effect.

The SVM model was finally used to develop an interactive web application. After the user enters the values for each metric and clicks “Submit,” the page will display the result of the prediction of the patient’s trajectory cluster, as well as the result of the analysis of the corresponding Shapley values (see Supplementary Figure 5). The details of the application can be found in https://xuxiaozhoushiny.shinyapps.io/application/.

## 4 Discussion

This study applied a purpose-driven cluster analysis, revealing three distinct clusters of motor symptom trajectories over a five-year follow-up in early PD patients. These clusters were characterized by different anthropometric features and showed significant differences in the progression of cardinal motor symptoms and the timing of reaching clinical milestones. The subtype classification, determined through longitudinal cluster analysis, may offer new insights into the dynamic heterogeneity of PD progression. Moreover, to identify trajectory clusters of new individuals in real-life clinical practice, we developed an ML model using baseline data and established an interactive web application based on this model.

Although several studies have attempted to categorize PD patients into various subtypes, most of these studies have been found to have significant methodological disadvantages and clinical applicability shortcomings (10, 18). Hypothesis-driven methods are important for answering specific research questions, but they categorize patients based on cross-sectional motor symptoms, compromising the stability of motor subtypes. On the other hand, the advantage of data-driven methods is that there are no *a priori* constraints, which may render the cluster result unreliable due to differences in variables and models, making the applicability unclear to researchers and users. Considering the respective characteristics of the two categories of subtyping studies, we believe that the purpose of use should be considered when developing and using PD subtypes.

The purpose-driven framework proposed by MDS provides guidance for defining PD subtypes and clarifying their application scenarios (18). The purpose-driven framework’s requirements for defining subtypes are: the ability to predict the progression of PD, the ability to predict the response to treatment, etc. However, the current technical and database conditions make it difficult to achieve a comprehensive approach that fully covers the heterogeneity of PD. Our research has advanced some objectives under the guidance of this framework. Firstly, accurately predicting the progression of PD is undoubtedly the top priority in clinical practice and research. For instance, in the prospective study based on the PREDICT-PD cohort conducted by Cristina’s team, it was possible to identify high-risk individuals for PD and longitudinally track the progression trajectory of their motor prodromal symptoms. However, this study was limited to assessments at the baseline and the 6th year of follow-up, which may affect the validity of the risk prediction model (26). This study utilizes a multi-time-point longitudinal follow-up data-driven definition as the basis for defining the prototype, an approach that may be more suitable for progressive diseases such as PD. In addition, Ren adopted the multivariate functional principal component analysis method to integrate the dynamic changes of multi-dimensional longitudinal indicators, and incorporated the extracted principal component features into the Cox model to construct the prognostic index (27). However, the 10 principal component features contained in this model lack established clinicopathological correlates. The subtype definition method based on the progression of motor symptoms in this study can avoid selection bias introduced by excessive variables. With the help of machine learning methods and interactive applications, clinical interpretation of clustering results and rapid prediction of new participants we can achieve.

Although the extent varies, motor symptoms in PD can lead to a loss of physical ability, changes in social life, alterations in relationships, and shifts in activity patterns (28). For instance, some individuals may feel troubled or even ashamed by exhibiting symptoms such as tremors or bradykinesia in public, leading to a withdrawal from social activities (29). Compared to the stable and intermediate clusters, the progressive cluster exhibits more severe motor and non-motor symptoms at baseline. Specifically, the progressive cluster exhibits more severe motor experiences in daily life, during motor examinations, and in the ability to perform daily activities. In terms of non-motor symptoms, the progressive cluster also performs worse than the stable cluster and the intermediate cluster in areas such as visuospatial function, executive function, and speed/attention. Furthermore, the progressive cluster has more severe impairments in olfactory and autonomic nervous functions compared to the stable cluster. Furthermore, the mean single-photon emission computed tomography (SPECT) striatal binding ratios for the progressive cluster are significantly lower than those for the other two clusters. This hierarchical ranking provides external validation for the trajectory clusters (13) and suggests the potential utility of SPECT imaging in assessing the progression of motor symptoms.

Moreover, living with PD means adapting to continuous losses. The disease’s progressive nature requires patients to continually adapt to the ongoing loss of daily living abilities as well, as face the additional challenges posed by its unpredictable trajectory. Coping with PD is thus never static, but an ongoing process of adapting to the circumstances of daily life with the disease (28). In terms of motor symptom progression, we found that the progression rates of cardinal motor symptoms varied across different clusters. The progressive cluster exhibited significant annual changes in tremor, rigidity, bradykinesia, and PIGD, with bradykinesia showing the fastest annual progression rate. Additionally, we observed that the progressive cluster had a higher incidence of reaching key clinical milestones within 5 years of follow-up. This cluster was more likely to experience disease progression, impairment in activities of daily living, sleep disturbances, and cognitive decline. Therefore, early identification of the progressive cluster can help PD patients adapt to gradual changes and maintain a positive outlook on life, which contributes to the long-term management of PD patients.

Currently, most typical clustering solutions present characteristics at the group level using mean values, which makes it impossible to directly categorize new individuals into different subgroups (13). Considering the clinical applicability, we used ML methods that have been widely used in medical research, with more comprehensive baseline clinical data, including demographic data, motor symptom assessment and non-motor symptom assessment to learn the characteristics of the cluster. The variables are typically evaluated at diagnosis, and are more accessible than the more costly cerebrospinal fluid and imaging studies. We also developed a user-friendly interactive web application for mobile devices. This application is based on the ML model, ensuring its foundation in our established classification method. After inputting patient characteristics, the tool identifies patient’s trajectory clusters online, which can assist in the long-term management of PD patients.

The current study has several limitations. Firstly, PD patients were untreated at baseline and received varying drug levels at follow-up, so we selected predictors of “off” status to reduce the impact of drugs. Secondly, the limitation of sample size leads to a relatively small number of subgroups after dividing patients into three trajectory groups. Furthermore, the trajectory clustering results of AMP PD cannot be used as rigorous external verification. Lastly, the web application is only trained based on PPMI data and have not yet been applied in real-world practices, which also limits the robustness of the model. Future research should use larger and more diverse PD cohorts to test and improve this model to enhance generalization and intra-cluster resolution.

## 5 Conclusion

Our study demonstrates that motor symptoms in PD patients exhibit dynamic progression over time. We identified three distinct trajectories in early PD patients, characterized by differing clinical features, clinical milestones and progression rates. To facilitate application, we developed an interactive web application based on an SVM model. These findings underscore the importance of understanding the dynamic nature of PD progression and highlight the critical role of early subtype identification for effective long-term management.

## Data Availability

Publicly available datasets were analyzed in this study. This data can be found at: http://ppmi-info.org/.
