# Supplementary material for: Identifying subtypes of longitudinal motor symptom severity trajectories in early Parkinson’s disease patients
Source: Front Neurol. 2025 Aug 20;16:1597132. doi: 10.3389/fneur.2025.1597132 (PMC12404927; doi:10.3389/fneur.2025.1597132)
Supplement: Supplementary file 1 [file Table_1.docx]

**Supplementary materials for**

**Identifying subtypes of** **longitudinal motor symptom severity trajectories in early Parkinson’s disease patients**

Xiaozhou Xu ^1,2, †^, Shushan Zhang ^3, †^, Chuanying Xu ^4, †^, Wei Zhang^4^, Hui Zhao ^4, 5^, Yumeng Liu ^1^, Shilei Zhai ^1^, Jie Zu ^4, 5, *^, Zhining Li ^6, *^, Lishun Xiao ^1, 7, 8, *^

1 Department of Biostatistics, School of Public Health, Xuzhou Medical University, Xuzhou, Jiangsu Province, 221004, China

2 Office of Hospital Quality and Safety Management, The First People’s Hospital of Lianyungang, Lianyungang, Jiangsu Province, 222061, China

3 Department of Ultrasound, the Fifth Affiliated Hospital of Sun Yat-sen University, Zhuhai, Guangdong Province, 519000, China

4 Department of Neurology, The Affiliated Hospital of Xuzhou Medical University, Xuzhou, Jiangsu Province, 221000, China

5 Department of Neurology, The First Clinical College, Xuzhou Medical University, Xuzhou, China

6 Department of Neurology, The Second Affiliated Hospital of Xuzhou Medical University, Xuzhou 221006, Jiangsu Province, China

7 Center for Medical Statistics and Data Analysis, Xuzhou Medical University, Xuzhou, Jiangsu Province, 221004, China

8 Key Laboratory of Human Genetics and Environmental Medicine, Xuzhou Medical University, Xuzhou, Jiangsu Province, 221004, China

† These authors have contributed equally to this work and share first authorship.

* Corresponding authors.

# Supplemental results


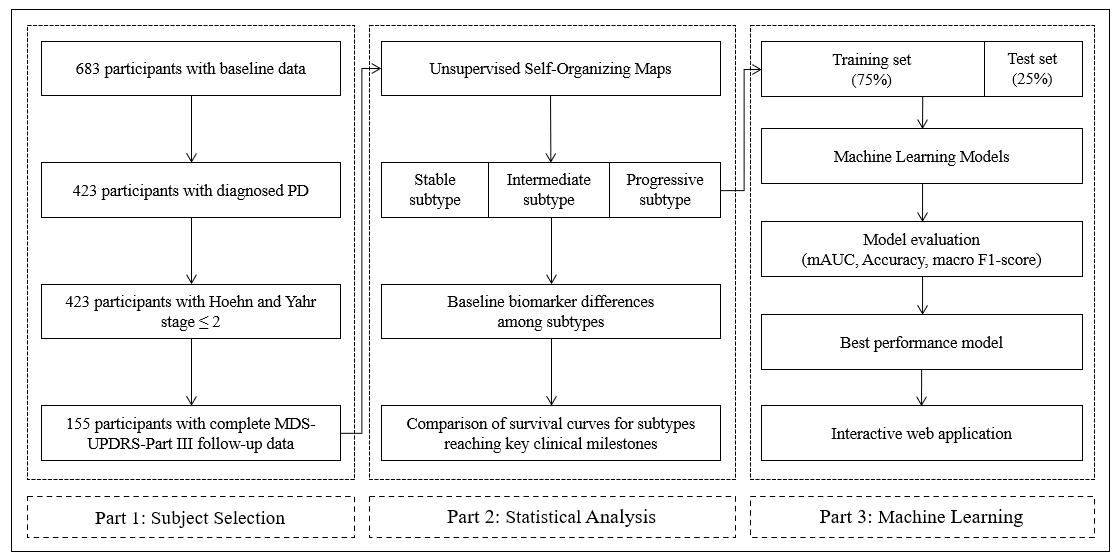


[**Supplementary**](https://www.sciencedirect.com/science/article/pii/S1353802023008866?dgcid=rss_sd_all&utm_source=researcher_app&utm_medium=referral&utm_campaign=RESR_MRKT_Researcher_inbound#appsec1) **Figure 1.** The detailed flow chart for enrolled 155 drug-naïve PD patients in PPMI.


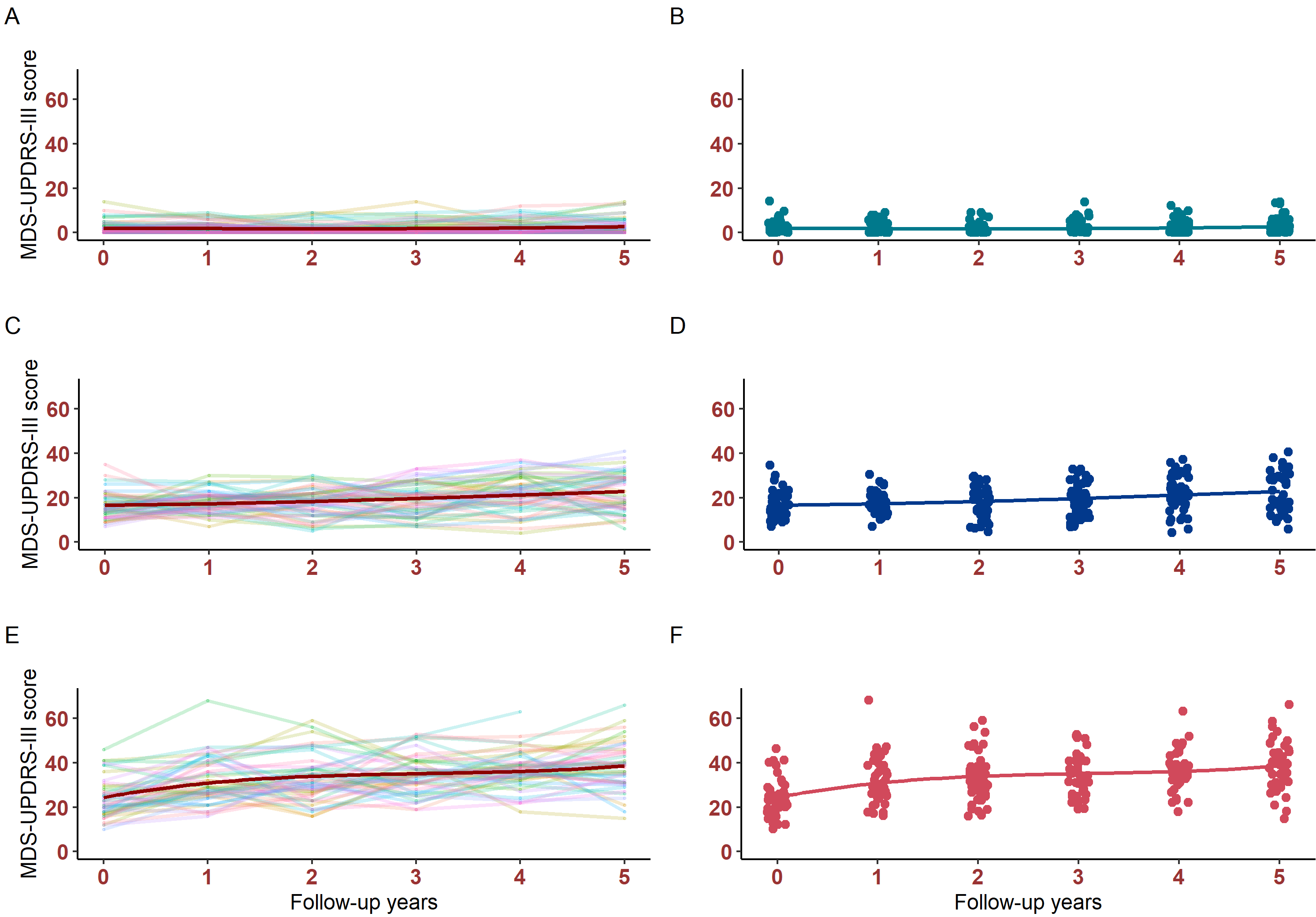


[**Supplementary**](https://www.sciencedirect.com/science/article/pii/S1353802023008866?dgcid=rss_sd_all&utm_source=researcher_app&utm_medium=referral&utm_campaign=RESR_MRKT_Researcher_inbound#appsec1) **Figure 2.** SOMs of MDS-UPDRS-Part III score trajectory per cluster among patients from AMP PD. Longitudinal line showing individual patient trajectories and mean progression trends for: Stable cluster (A), Intermediate cluster (C), and Progressive cluster (E). Corresponding scatter plots depicting score distribution and density for: Stable cluster (B), Intermediate cluster (D), and Progressive cluster (F).


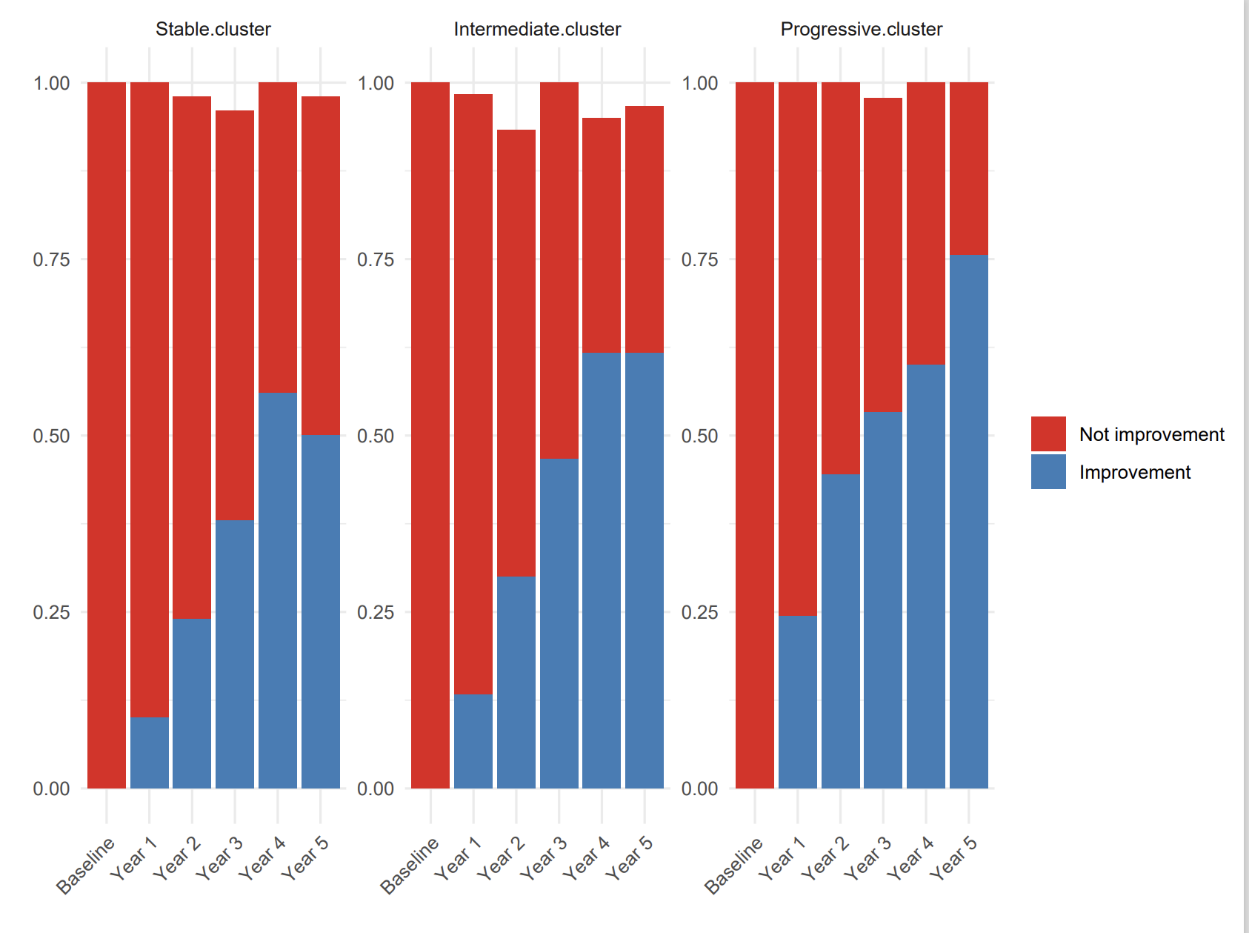


[**Supplementary**](https://www.sciencedirect.com/science/article/pii/S1353802023008866?dgcid=rss_sd_all&utm_source=researcher_app&utm_medium=referral&utm_campaign=RESR_MRKT_Researcher_inbound#appsec1) **Figure 3.** The proportion of MDS-UPDRS III improvement across different clusters after dopaminergic drug intake compared to OFF state. The improvement was defined as a change of ≥ 5 points in the MDS-UPDRS-Part III score. Some parts are incomplete due to the absence of open state data.


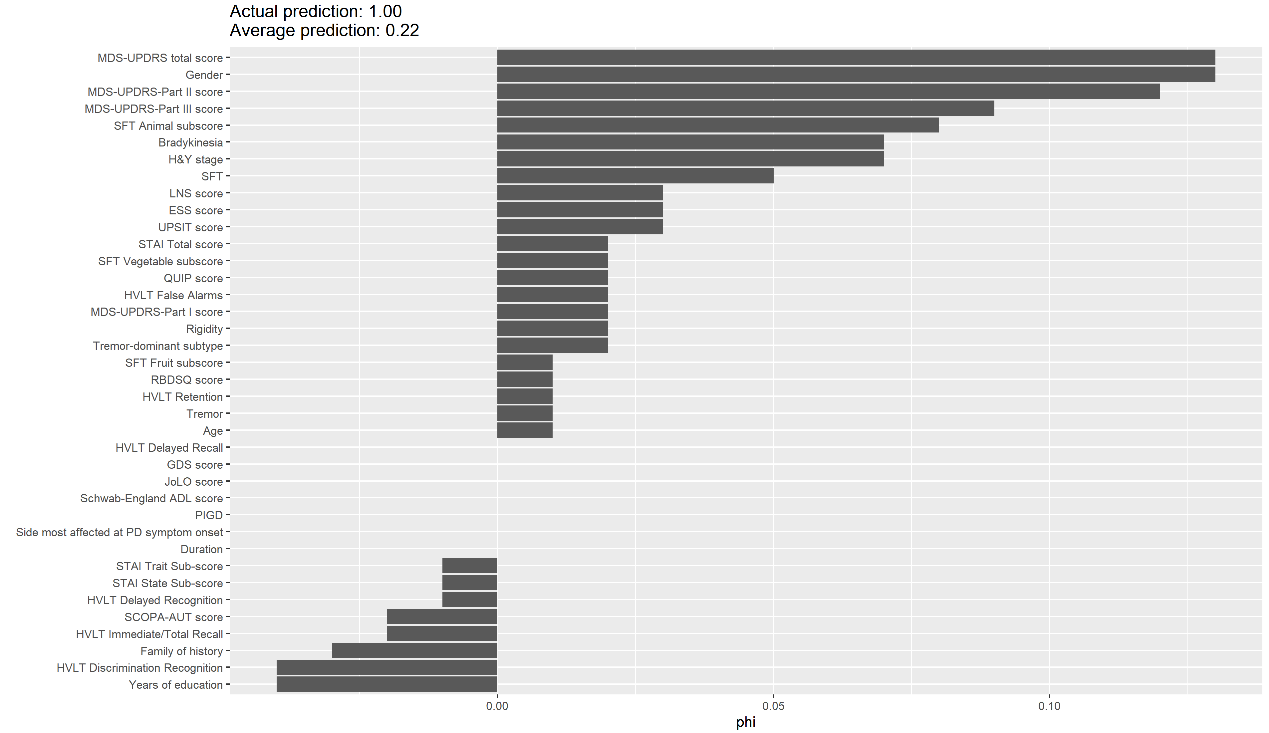


[**Supplementary**](https://www.sciencedirect.com/science/article/pii/S1353802023008866?dgcid=rss_sd_all&utm_source=researcher_app&utm_medium=referral&utm_campaign=RESR_MRKT_Researcher_inbound#appsec1) **Figure 4.** Shapley values for a PD patient in the PPMI dataset.


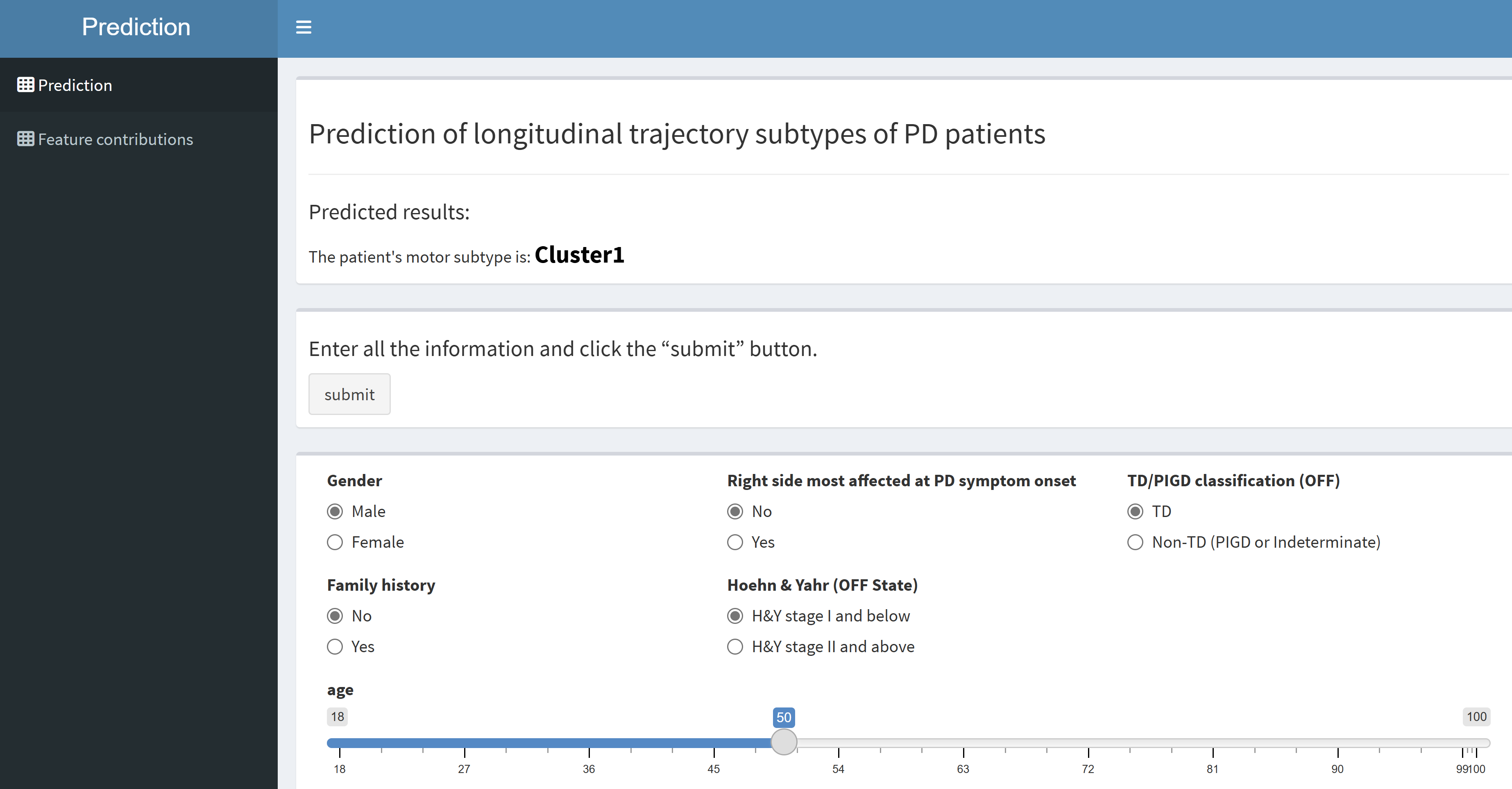


[**Supplementary**](https://www.sciencedirect.com/science/article/pii/S1353802023008866?dgcid=rss_sd_all&utm_source=researcher_app&utm_medium=referral&utm_campaign=RESR_MRKT_Researcher_inbound#appsec1) **Figure 5.** Screenshot of the interactive web application predicting trajectory cluster assignments for new PD patients.

[**Supplementary**](https://www.sciencedirect.com/science/article/pii/S1353802023008866?dgcid=rss_sd_all&utm_source=researcher_app&utm_medium=referral&utm_campaign=RESR_MRKT_Researcher_inbound#appsec1) **Table 1.** Description of the sample.

| Characteristics |  |
| --- | --- |
| Age, years | 61.51 [12.12] |
| Gender, n (%) |  |
| Male | 106 (68.39%) |
| Female | 49 (31.61%) |
| Age of onset, years | 59.98 [12.33] |
| Age of diagnosis, years | 61.26 [11.99] |
| Duration (months) | 3.87 [4.60] |
| Years of education | 16 [4] |
| MDS-UPDRS TOTAL score | 29 [17] |
| MDS-UPDRS-Part I score | 5 [5] |
| MDS-UPDRS-Part II score | 4 [6] |
| MDS-UPDRS-Part III score | 18 [10] |
| Rigidity | 3 [3] |
| Tremor | 5 [4] |
| bradykinesia | 6 [4] |
| PIGD | 1 [2] |
| H&Y stage, n (%) |  |
| Stage Ⅰ | 92 (59.35%) |
| Stage Ⅱ | 63 (40.65%) |
| Modified Schwab & England ADL score | 90 [10] |
| SCOPA-AUT score | 8 [6.5] |
| GDS score | 2 [2] |
| QUIP score | 0 [0] |
| STAI Total score | 61 [25] |
| STAI state subscore | 30 [15] |
| STAI trait subscore | 31 [11] |
| JoLO score | 14 [3] |
| HVLT Discrimination Recognition | 10 [2] |
| HVLT Immediate/Total Recall | 24.86±4.69 |
| HVLT Retention | 0.89 [0.26] |
| HVLT False Alarms | 1 [2] |
| HVLT Delayed Recall | 9 [3] |
| HVLT Delayed Recognition | 12 [1] |
| LNS score | 11 [3.5] |
| MOCA score | 28 [2] |
| UPSIT score | 23 [14] |
| ESS score | 5 [4] |
| RBDSQ score | 4 [4] |
| SDMT score | 43 [13] |
| SFT score | 48 [15.5] |
| SFT animal subscore | 21 [8] |
| SFT fruit subscore | 14 [5] |
| SFT vegetable subscore | 14.55±4.43 |
| DaTScan mean caudate SBR | 1.96±0.5 |
| DaTScan mean putamen SBR | 0.78 [0.30] |
| DaTScan mean striatum SBR | 1.33 [0.43] |
| CSF Aβ1–42 | 898.20 [498.01] |
| CSF α-synuclein | 1422.24 [701.3] |
| CSF t-Tau | 151.40 [70.10] |
| CSF p-Tau181 | 13.10 [5.32] |

Notes: Descriptive statistics for continuous variables are presented as median [IQR] or mean ± SD.

[**Supplementary**](https://www.sciencedirect.com/science/article/pii/S1353802023008866?dgcid=rss_sd_all&utm_source=researcher_app&utm_medium=referral&utm_campaign=RESR_MRKT_Researcher_inbound#appsec1) **Table 2.** Description of the AMP PD.

| Characteristics | Stable cluster  (N =45) | Intermediate cluster  (N =45) | Progressive cluster  (N =56) | *P*-value |
| --- | --- | --- | --- | --- |
| MDS-UPDRS total score | 5 [2.5] | 25 [7.5] | 39 [10] | **<0.001*^abc^*** |
| MDS-UPDRS-Part I score | 3 [2.125] | 5 [3] | 9 [3.5] | **<0.001*^abc^*** |
| MDS-UPDRS-Part II score | 0[0.5] | 3 [2] | 7 [4] | **<0.001*^abc^*** |
| MDS-UPDRS-Part III score | 1 [1.125] | 15 [4] | 23 [5] | **<0.001*^abc^*** |

Notes: Descriptive statistics for continuous variables are presented as median [IQR]. ***^a^*** Stable cluster is significantly different from intermediate cluster (Dunn’s method with *P*< 0.05). ***^b^*** Stable cluster is significantly different from progressive cluster (Dunn’s method with *P* < 0.05). ***^c^*** Intermediate cluster is significantly different from progressive cluster (Dunn’s method with *P* < 0.05).

# Supplemental methods

## PPMI exploratory biomarker analysis

The PPMI study website includes a repository of biomarker data, which were measured through various project protocols:

- DatScan SPECT imaging was measured at PPMI imaging centers according to the PPMI imaging protocol. Processing and calculation of striatal binding ratios (SBRs) were conducted at the Institute for Neurodegenerative Disorders (New Haven, CT, USA) according the methods as follows. SPECT raw projection data was imported to a HERMES (Hermes Medical Solutions, Skeppsbron 44, 111 30 Stockholm, Sweden) system for iterative (HOSEM) reconstruction. This was done for all imaging centers to ensure consistency of the reconstructions. Iterative reconstruction was done without any filtering applied. The HOSEM reconstructed files were then transferred to the PMOD (PMOD Technologies, Zurich, Switzerland) for subsequent processing. Attenuation correction ellipses where drawn on the images and a Chang 0 attenuation correction was applied images utilizing a site specific mu that was empirically derived from phantom data acquired during site initiation for the trial. Once attenuation correction was completed a standard Gaussian 3D 6.0 mm filter was applied. These files were then normalized to standard Montreal Neurologic Institute (MNI) space so that all scans were in the same anatomical alignment. Next the transaxial slice with the highest striatal uptake was identified and the 8 hottest striatal slices around it were averaged in to generate a single slice image. Regions of interest (ROI) were then place on the left and right caudate, the left and right putamen, and the occipital cortex (reference tissue). Count densities for each region were extracted and used to calculate striatal binding ratios (SBRs) for each of the 4 striatal regions. SBR is calculated as (target region/reference region)-1. Imaging data were presented as the regional specific binding ratio of the specific binding ratio by striatal sub-region. Mean caudate SBR was calculated as the sum of the right and left caudate SBRs divided by two. Mean putamen SBR was calculated as the sum of the right and left putamen SBRs divided by two. Mean striatum SBR was calculated as the sum of the right and left caudate and putamen SBRs divided by four.
- Aβ1-42, t-tau, and p-tau181 concentrations in cerebrospinal fluid (CSF) were measured using the Elecsys electro-chemi-luminescence immunoassays on the fully automated cobas e601 analyzer (Roche Diagnostics) at the University of Pennsylvania (Philadelphia, PA, USA). These analyses correspond to PPMI Project IDs 125 and 159.
- Alpha-synuclein concentrations in CSF were measured using an ELISA assay commercially available from BioLegend (San Diego, CA, USA). This analysis corresponds to PPMI Project ID 124.

More details are described on the PPMI data download website (<https://www.ppmi-info.org/access-data-specimens/download-data>).
